# Impact of Point-of-Care Decision Support Tool on Laboratory Screening for Comorbidities in Children with Obesity

**DOI:** 10.3390/children7070067

**Published:** 2020-06-27

**Authors:** Tara K. Kaufman, Natalie Gentile, Seema Kumar, Marian Halle, Brian A. Lynch, Valeria Cristiani, Karen Fischer, Rajeev Chaudhry

**Affiliations:** 1 Department of Family Medicine, Mayo Clinic, Rochester, MN 55905, USA; kaufman.tara@mayo.edu (T.K.K.); ngentile5988@gmail.com (N.G.); 2 Division of Pediatric Endocrinology and Metabolism, Mayo Clinic, 200 First Street SW, Rochester, MN 55905, USA; 3 Department of Health Sciences Research, Mayo Clinic, Rochester, MN 55905, USA; halle.marian@mayo.edu (M.H.); Fischer.karen@mayo.edu (K.F.); Chaudhry.rajeev@mayo.edu (R.C.); 4 Department of Pediatric and Adolescent Medicine, Mayo Clinic, Rochester, MN 55905, USA; lynch.brian@mayo.edu (B.A.L.); cristiani.valeria@mayo.edu (V.C.)

**Keywords:** pediatric obesity, diabetes mellitus, type 2, dyslipidemia, point-of-care systems, liver diseases, comorbidity

## Abstract

Background: Childhood obesity is associated with dyslipidemia, fatty liver disease, and type 2 diabetes. Expert guidelines recommend screening for these conditions in children with obesity. Aims and objectives: The objective of the study was to compare rates of laboratory screening for dyslipidemia, fatty liver disease, and type 2 diabetes in children with obesity prior to and following implementation of a point-of-care decision support tool. Methods: We performed a retrospective record review of children with body mass index (BMI) ≥95th percentile for age and gender (age 7–18 years) undergoing well-child/sports examination visits. Multivariable logistic regression models were used to adjust for patient and provider confounders. Results: There was no increase in the rates of screening following implementation of the point-of-care decision support tool. Tests were more likely to be recommended in children with severe obesity and in females. Conclusions: The implementation of a point-of-care decision support tool was not associated with improvement in screening rates for dyslipidemia, fatty liver disease, and type 2 diabetes for children with obesity. Further strategies are needed to improve rates of screening for obesity-related comorbid conditions in children with obesity.

## 1. Introduction

Childhood obesity is a major public health problem that has reached epidemic proportions [1]. According to the most recent data, 18.5% of school age children (6–11 years of age) and 20.6% of adolescents (12–19 years of age) in the United States have obesity [1]. Childhood obesity is associated with several comorbid conditions including dyslipidemia, fatty liver disease, prediabetes, and type 2 diabetes [2,3]. Obesity affects racial and ethnic minorities disproportionately and this has likely contributed to the increase in incidence of type 2 diabetes among youths of minority racial and ethnic groups over time [4]. Obesity, defined as body mass index (BMI) at or above the 95th percentile for age and gender, is the most common cause of liver disease in children [5]. Expert guidelines recommend screening children with obesity for dyslipidemia, fatty liver disease, and type 2 diabetes [3,6,7]. Screening for hyperlipidemia in children is advised in children 2 years of age or older with obesity every 1–3 years [8,9]. Screening for non-alcoholic fatty liver disease (NAFLD) is recommended starting between age 9 and 11 for all children with obesity with repeat measurements every 2–3 years if initial reports are normal [8,10]. Screening for type 2 diabetes is recommended in children with BMI in overweight or obese range at age 10 years or sooner if onset of puberty occurs before age 10 and if they have one or more risk factors for type 2 diabetes with repeat screening every 3 years [8,11]. The risk factors include family history of type 2 diabetes in a first or second degree relative, high risk race/ethnicity, maternal history of diabetes or gestational diabetes during the child’s gestation and signs of insulin resistance on physical examination or conditions associated with insulin resistance (acanthosis nigricans, hypertension, dyslipidemia, polycystic ovary syndrome or small for gestational age birth weight).

Various studies have demonstrated suboptimal rates of screening for these co-morbid conditions in children with obesity [12,13,14,15]. Electronic health record-based decision support has been shown to be effective in improving care in the pediatric setting for asthma [16], vaccinations [17], and prescribing patterns [18]. Computer assisted decision tools that alert providers to an elevated BMI and standardize pediatric weight management have been developed [19] and have been shown to improve identification, diagnosis, and counseling for overweight or obese children and adolescents [20,21,22,23]. There is limited data however on whether these tools improve laboratory screening for obesity-related comorbidities [22].

The objective of this study was to compare rates of laboratory screening for abnormalities in lipids, liver enzymes, and glucose in children with obesity prior to and following implementation of a point-of-care decision support tool in a primary care practice.

## 2. Methods

The target population for this study was all children aged 7 to 18 years seen in the primary care practice for a well-child visit with electronic consent for medical records research and a BMI ≥95th percentile for age and gender between January 2009 and December 2013. Severe obesity was defined as BMI ≥99th percentile for age and gender. The study was approved by the institutional review board at Mayo Clinic.

**Figure 1:**
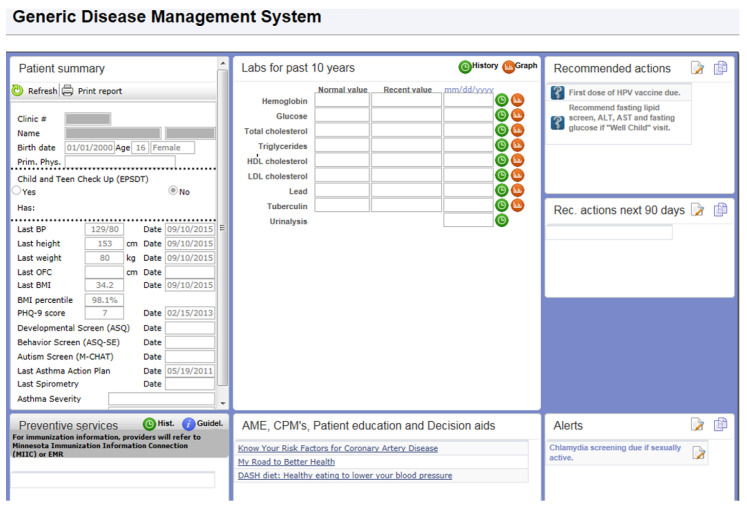
Decision support tool.

During the time of this study, Mayo Clinic’s electronic medical record was General Electric Centricity. Our clinical decision support system for preventive services and disease management was not fully developed for use in Centricity. To address this need, VitalHealth Software, a joint venture between Mayo Clinic and Netherlands-based Noaber Foundation, developed the Generic Disease Management System (GDMS) software. GDMS is a web-based application that uses General Electric Web Services and an MSQweb.net platform to retrieve patient vital statistics (such as blood pressure, weight, height, age, etc.) and other data elements required for decision making (Figure 1).

The GDMS included a rules-based application in which national guidelines for age- and sex-specific preventive services and for process and outcome measures for chronic conditions were coded. On the basis of data from Web services, the rules provided point-of-care decision support regarding the services that the patient needed at the time of his or her visit. For the current study, the clinical decision support for children with a BMI ≥85th percentile was developed in GDMS and introduced in all primary care clinics affiliated with Mayo Clinic, Rochester or Kasson, MN in September 2010. GDMS alerted the health care provider if the patient’s BMI percentile was in the overweight (BMI between 85th and 94th percentile) or obese range (≥95th percentile). If the patient was seen for a general medical examination or sports physical and was 10 years of age or older, and BMI was ≥95th percentile or between the 85th and the 95th percentile with a positive family history of diabetes and early or premature heart disease, then fasting lipids and glucose, hemoglobin A1C, aspartate transferase (AST) and alanine transferase (ALT) were recommended.

An independent data abstractor retrospectively electronically extracted data from the medical records of children between the ages of 7 and 18 years with BMI ≥95th percentile who presented for well-child/general medical examination/sports physical visits during 2009, 2011 or 2013. BMI was calculated from weight and height obtained at the same visit using the formula BMI = [weight in kg]/ [height (m)^2^]. If a child had been seen multiple times for a qualifying visit in the same year, only the first visit with weight and height information was considered. We chose the approach of counting the first visit during the year in order to maintain consistency and give similar weightage to characteristics of each patient regardless of the number of clinic visits during the year. Electronic medical records were searched to determine if relevant tests (lipid panel, AST, ALT, fasting plasma glucose, and HbA1c) were performed during the study period. Children in whom these laboratory tests had been performed in the five years prior to the visit were excluded from the study.

Laboratory tests were considered performed if they were done within 90 days before or after the qualifying visit appointment. Other data abstracted from the electronic medical records included patient sex, age (years), insurance type (government vs. private), need for interpreter, provider affiliation (pediatrics vs. family medicine), and provider type (resident physician, staff physician, nurse practitioner/physician assistant).

A subset of randomly selected charts was manually reviewed (25 charts per year) by two independent data extractors (T.K. and M.H.) to confirm the reliability of the electronic search.

Records from the year 2009 were reviewed to assess rates of laboratory screening for obesity-related complications among obese children and adolescents prior to implementation of the point-of-care decision support tool and those from 2011 and 2013 were reviewed for screening rates after implementation of the tool.

## 3. Statistical Methods

Baseline subject characteristics for each year were summarized using frequency percentages. The primary outcome of interest was whether there was a difference in the frequency of laboratory tests performed before (2009) and after (2011 and 2013) implementation of the decision support tool. The odds ratios of having at least one test ordered for both the univariate analysis and the multivariate analysis were calculated using a generalized estimating equation (GEE) logistic regression model. Gender, race, specialty, age range, insurance, provider type, and obesity status were all adjusted for in the model. The GEE model was used due to the possibility that the same child had multiple entries for 2009, 2011, and 2013. A Cochran–Armitage test for trend was used to determine a significant trend in the tests ordered for each individual test over the three time points. In all cases, a two-tailed *p*-value of less than 0.05 was considered significant. Statistical analysis was done using SAS statistical software (SAS version 9.4; SAS Institute Inc.).

## 4. Results

The number of patients whose health care visit records were eligible for review was 342 in the year 2009, 366 in the year 2011, and 379 in the year 2013. Table 1 shows the descriptive characteristics of the study population. The majority of patients were White (78.2%), had obesity that was not severe (85.2%) and had commercial insurance (80.7%). Approximately 60.5% were between 11–14 years of age and slightly more than half (56.1%) were seen by a staff physician (56.1%). There were no statistically significant differences in demographic and anthropometric characteristics among study participants from the three years.

Table 2 details the patient and provider characteristics associated with testing. The full model adjusted for the covariates of year, gender, race, age, specialty, insurance type, provider type, and obesity status. After adjusting for these covariates, testing was significantly more likely performed for females relative to males (odds ratio (OR) = 1.95, *p* = 0.001), for those older than 14 years (OR = 1.61, *p* = 0.001), and those with severe obesity compared to those with non-severe obesity (OR = 2.54, *p* < 0.001). Laboratory screening was more likely performed in children seen by pediatricians relative to those seen by family medicine providers (OR = 4.1, *p* <0.001).

Table 3 defines the tests performed within 90 days of clinical note date. There was no significant difference in rates of testing for abnormalities in lipids, liver enzymes or glucose following the implementation of the decision support tool.

The vast majority of patients had no tests performed (85.9%) and a small proportion (8.7%) had all five tests performed.

## 5. Discussion

We examined the impact of a point-of-care decision support tool in a primary care practice on rates of recommended laboratory screening for abnormalities in lipids, liver enzymes, and glucose in children and adolescents with obesity. We found no increase in the rates of screening for these obesity-related comorbidities following implementation of the point-of-care decision support tool. To our knowledge, this is the first study to examine the impact of implementation of a point-of-care decision tool without any additional intervention such as provider education or addition of embedded order sets on rates of screening for obesity-related comorbid conditions in children with obesity in primary care.

Our findings of no changes in rates of screening for dyslipidemia and diabetes following implementation of the decision support tool are in contrast to those of Shaikh and colleagues who demonstrated an increase in the rates of screening for dyslipidemia and diabetes from 17% to 27% after implementation of a decision support tool [22]. These differences may be related to variances in the specific components of the decision support tool. The decision support tool in the study by Shaikh and colleagues had an embedded smart set with links to orders for the recommended laboratory tests, thereby making it easier for the providers to order the tests immediately after they see the alert. The decision support tool in our study, on the other hand, did not have any links to the recommended tests. Unlike our study, the providers in the other study received a one hour training session which included an update on national recommendations for the evaluation and management of pediatric obesity, information on the alert, as well as the associated clinical decision-support tools. Another difference was with regards to the characteristics of the patients. While our study included children between ages 7 and 18 years, Shaikh and colleagues studied children between 2 and 18 years of age.

A systematic review showed that point-of-care reminders were generally associated with only small-to-modest changes in clinician behavior [24]. One factor that is likely contributing to the low rates of screening for dyslipidemia despite the decision support tool is the lack of evidence on the benefits of screening for dyslipidemia during childhood [25]. Another likely factor is alarm fatigue with an increase in the number of alerts in electronic health records over the years. Qualitative interviews of pediatric providers at a primary care network of Cambridge Health Alliance in Massachusetts suggested that the majority of providers did not feel that the electronic supports were helpful [26]. The support tools were perceived as interfering with the workflow and engagement with patients. Other barriers include inadequate knowledge [27,28], insufficient reimbursement [29,30], lack of resources [27] and lack of time [29,31]. In a cross-sectional, self-administered mail survey which queried members of the North Carolina Pediatrics Society and the American Academy of Pediatrics who were practicing routine care, only a minority (12%) reported high self-efficacy in obesity management, thereby highlighting the need for better education of providers regarding obesity management and screening for comorbid conditions [32].

Another reason for the low rates of screening is the lack of buy in from many patients and families themselves who would prefer modifying dietary and physical habits prior to getting the tests drawn. Therefore, while providers may have recommended and ordered screening tests, families may have elected to not proceed with getting the testing completed with the hope of optimizing their lifestyle before screening.

We found higher rates of screening in females than in males. Similar findings were reported by Benson and colleagues [12]. The higher rates of screening in females may be related to 1.3–1.7-fold higher chance of development of type 2 diabetes during adolescence in females relative to males. No differences in screening rates were however reported in two other studies [13,14].

The results of our study suggest that introduction of a decision support tool merely is not sufficient to change provider behavior and adoption of expert guidelines. It needs to be accompanied by education of the providers regarding the tool itself and the expert guidelines on recommended care of specific conditions. Additionally, a smart set with links to recommended tests or consultations should be considered as part of the decision support tool to facilitate change in practice by the providers. Timely feedback to the providers regarding their practice habits may be another strategy to ensure success of the decision support tool in terms of improving adherence to national guidelines.

One strength of this study was that we examined if there was an association between patient’s socioeconomic status and screening for obesity-related comorbid conditions. We used the presence of government-issued insurance (Medicaid) as a surrogate measure of the socioeconomic status. Of note, we did not find Medicaid insurance status to be associated with rates of screening.

A limitation of the current study is that we considered screening performed only if the patient had completed the laboratory tests. It is certainly possible that tests may have been ordered by the providers. However, the patient or family may have chosen to not get the test done. Another limitation was the low proportion of children from racial and ethnic minorities, which are disproportionally affected by comorbid conditions including type 2 diabetes and fatty liver disease. Therefore, the results may not be generalizable to a diverse population as the study was conducted at a medical center in the Midwestern United States that serves a predominantly White population.

## 6. Conclusions

The implementation of a point-of-care decision support tool was not associated with improvement in screening for dyslipidemia, fatty liver disease, and type 2 diabetes in children with obesity. Further strategies are needed in conjunction with decision support tools in order to improve rates of screening for obesity-related comorbid conditions in children with obesity.

## Figures and Tables

**Table 1:** Descriptive characteristics of study participants.

	Total (*N* = 1087)	2009 (*N* = 342)	2011 (*N* = 366)	2013 (*N* = 379)
Gender				
Male	660 (60.7%)	202 (59.1%)	231 (63.1%)	227 (59.9%)
Female	427 (39.3%)	140 (40.9%)	135 (36.9%)	152 (40.1%)
Race				
Black	77 (7.1%)	18 (5.3%)	23 (6.3%)	36 (9.5%)
White	850 (78.2%)	271 (79.2%)	288 (78.7%)	291 (76.8%)
Unknown	160 (14.7%)	53 (15.5%)	55 (15.0%)	52 (13.7%)
Specialty				
Family Medicine	559 (51.4%)	171 (50.0%)	198 (54.1%)	190 (50.1%)
Pediatrics	528 (48.6%)	171 (50.0%)	168 (45.9%)	189 (49.9%)
Age group				
7–10 years	89 (8.2%)	28 (8.2%)	29 (7.9%)	32 (8.4%)
11–14 years	658 (60.5%)	202 (59.1%)	218 (59.6%)	238 (62.8%)
15–18 years	340 (31.3%)	112 (32.8%)	119 (32.5%)	109 (28.8%)
Insurance				
Commercial	877 (80.7%)	285 (83.3%)	293 (80.1%)	299 (78.9%)
Medicaid	176 (16.2%)	49 (14.3%)	60 (16.4%)	67 (17.7%)
Other	34 (3.1%)	8 (2.3%)	13 (3.6%)	13 (3.4%)
Provider Type *^†^				
Nurse Practitioner	251 (23.2%)	54 (15.8%)	112 (30.8%)	85 (22.6%)
Resident	224 (20.7%)	74 (21.7%)	73 (20.1%)	77 (20.5%)
Staff	606 (56.1%)	213 (62.5%)	179 (49.2%)	214 (56.9%)
Obesity Status				
Non Severe	926 (85.2%)	297 (86.8%)	316 (86.3%)	313 (82.6%)
Severe	161 (14.8%)	45 (13.2%)	50 (13.7%)	66 (17.4%)

* There were 6 missing values for the provider type variable. ^†^ The frequency of provider type was significantly different between years (Pearson Chi-squared test *p*-value < 0.001).

**Table 2:** Patient and provider factors associated with performance of screening tests.

	Univariate Analysis		Multiple Regression Analysis	
	Odds Ratio (95% CI)	*p*-Value *	Odds Ratio (95% CI)	*p*-Value *
Year				
2009	ref		ref	
2011	1.09 (0.73, 1.64)	0.675	1.17 (0.75, 1.83)	0.487
2013	0.82 (0.53, 1.26)	0.362	0.83 (0.52, 1.33)	0.438
Gender				
Male	ref		ref	
Female	1.91 (1.35, 2.70)	0.0002	1.95 (1.33, 2.87)	0.001
Race				
White	ref		ref	
Black	1.05 (0.54, 2.04)	0.890	0.58 (0.25, 1.32)	0.192
Unknown	1.17 (0.73, 1.87)	0.518	1.11 (0.66, 1.87)	0.700
Specialty				
Family Medicine	ref		ref	
Pediatrics	4.14 (2.79, 6.14)	<0.001	5.14 (3.34, 7.92)	<0.001
Age Group				
7–10	0.32 (0.12, 0.90)	0.031	0.21 (0.07, 0.63)	0.005
11–14	ref		ref	
15–18	1.61 (1.14, 2.30)	0.008	1.93 (1.32, 2.84)	0.001
Insurance				
Commercial	ref		ref	
Medicaid	1.33 (0.87, 2.05)	0.192	1.16 (0.68, 1.99)	0.588
Other	0.17 (0.02, 1.23)	0.080	0.18 (0.03, 1.29)	0.088
Provider Type				
Staff	ref		ref	
NP	1.46 (0.97, 2.19)	0.071	1.32 (0.83, 2.10)	0.234
Resident	1.32 (0.85, 2.03)	0.213	1.11 (0.69, 1.78)	0.671
Obesity Status				
Moderate	ref		ref	
Severe	2.18 (1.45, 3.29)	0.0002	2.54 (1.61, 4.01)	<0.001

* Chi-squared test. An outcome of 1 means that at least 1 test was ordered compared to not having any tests ordered.

**Table 3:** Proportion of patients with laboratory tests performed.

	Total (*N* = 1087)	2009 (*N* =342)	2011 (*N* = 366)	2013 (*N* = 379)	*p*-Value *
Glucose					
Yes	135 (12.4%)	43 (12.6%)	53 (14.5%)	39 (10.3%)	0.338
No	952 (87.6%)	299 (87.4%)	313 (85.5%)	340 (89.7%)	
A1C					
Yes	17 (1.6%)	6 (1.8%)	4 (1.1%)	7 (1.9%)	1.00
No	1070 (98.4%)	336 (98.3%)	362 (98.9%)	372 (98.2%)	
ALT					
Yes	110 (10.1%)	35 (10.2%)	51 (13.9%)	24 (6.3%)	0.073
No	977 (89.9%)	307 (89.8%)	315 (86.1%)	355 (93.7%)	
Triglycerides					
Yes	131 (12.1%)	44 (12.9%)	49 (13.4%)	38 (10.0%)	0.253
No	956 (88.0%)	298 (87.1%)	317 (86.6%)	341 (90.1%)	
HDL					
Yes	129 (11.9%)	44 (12.9%)	48 (13.1%)	37 (9.8%)	0.205
No	958 (88.1%)	298 (87.1%)	318 (86.9%)	342 (90.2%)	
LDL					
Yes	129 (11.9%)	44 (12.9%)	48 (13.1%)	37 (9.8%)	0.205
No	958 (88.1%)	298 (87.1%)	318 (86.9%)	342 (90.2%)	
Any Testing Performed					
Yes	153 (14.1%)	50 (14.6%)	57 (15.6%)	46 (12.1%)	0.335
No	934 (85.9%)	292 (85.4%)	309 (84.4%)	333 (87.9%)	

* Cochran–Armitage test for trend. ALT = alanine transferase; HDL = high-density lipoprotein; LDL = low-density lipoprotein.
